# Prévalence du burnout en milieu d'anesthésie réanimation dans le centre tunisien

**DOI:** 10.11604/pamj.2018.31.111.10739

**Published:** 2018-10-12

**Authors:** Salah Mhamdi, Mohamed Said Nakhli, Mohamed Kahloul, Nadia Latrech, Mohamed Ben Rejeb, Majdi Khadhraoui, Ajmi Chaouch, Walid Naija

**Affiliations:** 1 Service d'Anesthésie Réanimation, CHU Sahloul Sousse, Faculté de Médecine Ibn Al Jazzar, Sousse, Tunisie; 2 Service d'Epidémiologie, CHU Sahloul Sousse, Faculté de Médecine Ibn Al Jazzar, Sousse, Tunisie

**Keywords:** Burnout, anesthésie réanimation, prévalence, Burnout, anesthesia and intensive care unit, prevalence

## Abstract

**Introduction:**

l'épuisement professionnel serait particulièrement préoccupant en milieu d'anesthésie réanimation. En plus de ses répercussions socio-économiques, il altère la qualité des soins prodigués et le pronostic des malades. Notre but est d'évaluer sa prévalence chez le personnel d'anesthésie-réanimation dans le centre tunisien.

**Méthodes:**

il s'agit d'une enquête multicentrique transversale réalisée au sein des services d'anesthésie-réanimation dans les sept centres hospitalo-universitaires du centre tunisien et portant sur tout le personnel médical et paramédical consentant. L'instrument de mesure utilisé est le *Maslach burnout Inventory*.

**Résultats:**

deux cent quatre-vingt-trois personnes ont participé à l'étude (72,19%). L'âge moyen était de 40,2 ± 9,38 ans avec une prédominance féminine. L'analyse de l'échelle de Maslach a révélé que 94,71% des participants étaient concernés par le burnout. Les scores moyens d'épuisement émotionnel, de dépersonnalisation et d'accomplissement professionnel étaient respectivement de 28,65 ± 11,92; 8,62 ± 6.65 et 34,58 ± 8,07. Un niveau élevé à modéré de burnout a été trouvé respectivement dans 13,3% et 26,2% des cas. Un niveau bas a été trouvé dans 55,21% des cas. Les répercussions du burnout sont dominées par les conduites additives (52,65%) et les idées suicidaires (4,59%).

**Conclusion:**

le burnout apparaît de plus en plus comme une réalité palpable chez le personnel d'anesthésie réanimation. Ses conséquences sont graves aussi bien sur le plan individuel que social.

## Abstract (en)

**Introduction:**

burnout is a particular cause of concern in Anesthesia and Intensive Care Units. In addition to its socio-economic impact, it alters the quality of care and patients prognosis. This study aims to assess its prevalence among the staff members of the Tunisian Anesthesia and Intensive Care Units.

**Methods:**

we conducted a multicenter cross-sectional study in the Anesthesia and Intensive Care Units of seven Tunisian University Hospitals. The study included the medical and paramedical staff who gave consent. The measuring instrument used was the Maslach burnout Inventory.

**Results:**

the study included 283 staff members (72.19%). The average age of subjects was 40.2 ± 9.38 years, with a female predominance. Maslach scale revealed that 94.71% of the participants had burnout. The mean emotional exhaustion score, depersonalization score and professional achievement score were 28.65 ± 11.92; 8.62 ± 6.65 and 34.58 ± 8.07 respectively. High to moderate burn-out level were found in 13.3% and 26.2% of cases respectively. Burn-out effects were dominated by additive behaviors (52.65%) and suicidal ideations (4.59%).

**Conclusion:**

burnout is becoming more and more a tangible reality for the staff members of the Anesthesia and Intensive Care Units, engendering serious social and personal consequences.

## Introduction

Le travail est ce par quoi l'homme transforme ce qui l'entoure pour satisfaire ses besoins. Cependant, et paradoxalement à cette satisfaction désirée, on parle de plus en plus de la souffrance au travail. En effet, depuis le début des années 90, la fréquence des problèmes de santé psychologique liés au travail augmente de façon alarmante. Ces problèmes incluent essentiellement l'épuisement professionnel, la dépression, le stress post-traumatique et les troubles anxieux [1, 2]. L'épuisement professionnel ou burnout est un concept introduit pour la première fois en 1974 par le psychanalyste américain Freudenberger pour décrire l'état d'épuisement et de vide interne observé chez les individus impliqués dans une relation d'aide et plus particulièrement les professions médicales et paramédicales [3-5]. De multiples définitions se sont succédées [3, 6-8], mais finalement c'est la description de Maslach et Jackson qui serait la plus répandue [9, 10]. Cette définition utilise le *Maslash Burnout Inventory* comme instrument de mesure permettant à la fois de faire le diagnostic de cette pathologie et d'évaluer sa sévérité [11, 12]. Dans le domaine de la santé, le burnout serait particulièrement préoccupant notamment pour le personnel d'anesthésie réanimation qui prend en charge en permanence des pathologies vitales [13, 14]. Pour ce personnel, la mission principale est de sauver des vies et de soulager les souffrances des autres. L'accomplissement de cette mission n'est pas du tout facile quand on est confronté à des contraintes majeures telle que le rythme excessif de travail, la diversité et la gravité des pathologies, l'urgence et la rapidité d'intervention avec le risque des conséquences dramatiques en cas d´erreur et les conflits éthiques récurrents [15, 16]. Il est donc particulièrement intéressant d'étudier le burnout en milieu d'anesthésie réanimation non seulement à cause de ses répercussions économiques et sociales, mais également à cause de son retentissement sur la qualité des soins prodigués et par conséquent sur la prise en charge des malades [15, 17]. Cependant, et malgré les conséquences graves du burnout en milieu d'anesthésie réanimation, très peu d'études se sont intéressées à étudier ce problème notamment dans les pays maghrébins. Pour cela, nous avons réalisé cette étude dans les Centres Hospitalo-Universitaires (CHU) tunisiens afin d'évaluer la prévalence et déterminer le profil épidémiologique du syndrome d'épuisement professionnel auprès des professionnels de la santé en milieu d'anesthésie-réanimation.

## Méthodes

Il s'agit d'une enquête multicentrique transversale par questionnaire réalisée sur une période d'un mois (du 1^er^au 31 décembre 2014) au sein des services d'anesthésie-réanimation dans les sept CHU du centre tunisien: l'hôpital Fattouma Bourguiba de Monastir, la maternité de Monastir, l'hôpital Sahloul de Sousse, l'hôpital Farhat Hached de Sousse, l'hôpital Al Aghaliba de Kairouan, l'hôpital Ibn Jazzar de Kairouan et l'hôpital Taher Sfar de Mahdia. Les médecins anesthésistes-réanimateurs (MAR), les infirmiers anesthésistes diplômés d'état (IADE) et les infirmiers diplômés d'état (IDE) exerçant en tant que titulaires en milieu d'anesthésie-réanimation dans les établissements publics ont constitué la population cible de notre étude. Ceux qui étaient en congé annuel ou en arrêt maladie pendant la période d'étude ont été exclus. Nous avons utilisé le Maslach burnout Inventory comme seul instrument de mesure et ce pour garantir le maximum de participation et de concentration lors du remplissage du questionnaire. Cet instrument est un questionnaire constitué de 22 items, évaluant trois dimensions: l'épuisement émotionnel, la dépersonnalisation et l'accomplissement professionnel. Chaque item est coté selon une échelle de six points où 0 correspond à la réponse « jamais » et 6 à « tous les jours ». Le score attribué à chaque dimension correspond à la somme des cotations données à ses différents items et permet de classer cette dimension selon trois niveaux de sévérité à savoir bas, modéré ou sévère.

L'épuisement émotionnel est considéré comme bas si le score est inférieur à 17, modéré si le score est compris entre 18 et 29 et comme élevé si le score est supérieur à 30. Pour la dépersonnalisation, elle est considérée comme basse si le total est inférieur à 5, modérée pour un total compris entre 6 et 11 et élevée pour un total supérieur à 12. Enfin, l'altération du sentiment d'accomplissement professionnel est considéré comme bas pour un score supérieur à 40, modéré pour un score compris entre 34 et 39 et élevé pour un score inférieur à 33. Ces différentes dimensions nous ont permis de classer le burnout selon trois niveaux de sévérité. Un niveau élevé est défini par une atteinte sévère et complète des trois dimensions. Un niveau modéré est défini par deux dimensions pathologiques. Un niveau bas est défini par une seule dimension pathologique. Les participants ont été contactés directement sur leurs lieux d'exercice après obtention d'une autorisation des chefs de service. La liste exhaustive du personnel concerné a été récupérée au sein du secrétariat de chaque service. Le consentement des participants a été obtenu oralement avant la passation des questionnaires. Suite à une information claire et précise expliquant les objectifs de l'étude et assurant la confidentialité et l'anonymat des données, les questionnaires ont été distribués par le médecin enquêteur. Un délai de deux semaines était attendu pour les réponses. Les questionnaires non rendus ont été considérés comme des refus indirects qui ont été respectés. Les données ont été saisies et traitées par le logiciel SPSS dans sa version 21.00. Les variables qualitatives ont été exprimées en pourcentage. Les variables quantitatives ont été exprimées en moyenne avec écart type.

## Résultats

Nous avons distribué 392 questionnaires dont 283 ont été récupérés, ce qui correspond à un taux de réponse de 72,19%. La majorité des participants (57,95%) était des IADE. L'âge moyen dans notre population était de 40,2 ± 9,38 ans avec une nette prédominance féminine dont en témoigne un SR de 0,48. Le tiers des participants avait une ancienneté professionnelle dépassant les 15 ans. La majorité était mariée (83% des cas) avec au moins 3 enfants à charge dans 37,8% des cas. Les caractéristiques sociodémographiques et professionnelles de notre population sont résumées dans le Tableau 1. L'analyse de l'échelle de Maslach a révélé que 94,71% des participants étaient concernés par le burnout puisqu'ils présentent au moins une dimension pathologique. Les scores moyens d'épuisement émotionnel, de dépersonnalisation et d'accomplissement professionnel étaient respectivement de 28,65 ± 11,92 ; 8.62 ± 6.65 et 34,58 ± 8,7. Un niveau élevé de burnout a été trouvé dans 13,3% des cas. Un niveau modéré a été trouvé dans 26,2% des cas. Un niveau bas a été trouvé dans 55,21% des cas. Le burnout dans sa forme sévère a touché surtout les infirmiers diplômés d'état (IDE). Dans ce groupe, la dimension la plus altérée est l'épuisement émotionnel. La sévérité du burnout et de ses différentes dimensions en fonction du statut professionnel est résumée dans le Tableau 2. Quant aux répercussions du burnout, elles dépendent surtout de son importance. Elles sont dominées par les conduites additives (52,65%) et les idées suicidaires (4,59%). Elles s'observent surtout chez les IDE. Ces répercussions sont détaillées dans le Tableau 3.

**Tableau 1:** caractéristiques sociodémographiques

	MAR n=35	IADE n=164	IDE n=84	Total n= 283
**Age moyen (ans)**	40,83 ± 7, 48	42,84 ± 8,6	34,79 ± 9,3	40,20 ± 9.38
**Sex Ratio**	2.88	0.23	0.71	0.48
**Etat civil**				
Célibataire	3 (8.57%)	7 (4.26%)	29 (34.52%)	39 (13.78%)
Marié	31 (88.57%)	151 (92.07%)	53 (63.09%)	235 (83.03%)
Divorcé	1 (2.85%)	5 (3.04%)	1 (1.19%)	7 (2.47%)
Veuf	0	1 (0.61%)	1 (1.19%)	2 (0.7%)
**Nbre d’enfant**				
0	5 (14.28%)	29 (17.68%)	39 (46.42%)	73 (25.79%)
1 à 2	19 (54.28%)	58 (35.36%)	26 (30.95%)	103 (36.39%)
≥ 3	11 (31.42%)	77 (46.95%)	19 (22.61%)	107 (37.80%)
**Ancienneté**				
≤ 5 ans	12 (34.28%)	34 (20.73%)	42 (50%)	88 (31.09%)
6 à 15	13 (37.14%)	40 (24.39%)	30 (35.71%)	83 (29.32%)
> 15	10 (28.57%)	90 (54.87%)	12 (14.28%)	112 (39.57%)
**Horaires de travail**				
Jour	27 (77.14%)	141 (85.97%)	40 (47.61%)	208 (73.49%)
Nuit	0	10 (6.09%)	14 (16.66%)	24 (8.48%)
Posté	8 (22.85%)	13 (7.92%)	30 (35.71%)	51 (18.02%)
**Heures par semaine**				
<35	5 (14.28%)	25 (15.24%)	10 (11.9%)	40 (14.13%)
35 à 45	21 (60%)	131 (79.87%)	68 (80.95%)	220 (77.73%)
>45	9 (25.71%)	8 (4.87%)	6 (7.14%)	23 (8.12%)
**Taux de participation**	88.88	67.74	70.68	72.19

**Tableau 2:** sévérité du burn out et de ses différentes dimensions en fonction du statut professionnel

	MAR n=35	IADE n=164	IDE n=84	Total n= 283
**Epuisement professionnel**				
Bas	25.71%	33.74%	26.5%	55.21%
Modéré	54.25%	53.26%	47%	26.2%
Elevé	2.9%	8.1%	25.3%	13.3%
Pas de burn out	17.14%	4.9%	1.2%	5.29%
**Epuisement émotionnel**				
Bas	42.85%	18.29%	8.33%	18.37%
Modéré	34.28%	33.53%	27.38%	31.80%
Elevé	20%	46.95%	63.09%	48.40%
**Dépersonnalisation**				
Bas	42.85%	42.07%	11.90%	33.21%
Modéré	37.14%	33.53%	32.14%	33.56%
Elevé	17.14%	22.56%	54.76%	31.44%
**Accomplissement personnel**				
Bas	37.14%	31.09%	23.80%	29.68%
Modéré	28.57%	31.70%	27.38%	30.03%
Elevé	31.42%	35.97%	47.61%	38.86%

**Tableau 3:** les répercussions du burn out

	MAR n=35	IADE n=164	IDE n=84	Total n= 283
Heures de sommeil par jour	6.48	6.89	6.71	6.78
Arrêt maladie	1 (2.85%)	16 (9.75%)	9 (10.71%)	26 (9.18%)
Absentéisme	6 (17.14%)	44 (26.82%)	32 (38.09%)	82 (28.97%)
Conduites addictives	15 (42.85%)	82 (50%)	52 (61.9%)	149 (52.65%)
Idées suicidaires	1 (2.85%)	7 (4.26%)	5 (5.95%)	13 (4.59%)

## Discussion

Le burnout est une pathologie particulièrement préoccupante en milieu d'anesthésie réanimation. En effet, malgré un niveau de stress anormalement élevé, le personnel se trouve souvent dans l'obligation de prendre en charge des malades en détresse sans qu'il y ait le moindre reproche [15, 17]. Les conséquences de toute erreur ne peuvent être que lourdes et dramatiques. Les répercussions du burnout dans cet environnement sont donc aussi graves pour le personnel que pour les malades et la société. Cependant, et du moins dans les pays maghrébins, très peu d'efforts sont fournis afin d'étudier ce fléau et lui chercher des solutions [17]. Pour cela, nous avons mené cette étude multicentrique dans sept établissements hospitalo-universitaires du centre tunisien. Le taux de participation était de 72,19% permettant ainsi des résultats assez fiables. L'étude a été réalisée sur une période courte afin de palier à la fluctuation des données en fonction du temps. Le choix de l'hiver pourrait surestimer la fréquence du burnout à cause d'une probable confusion avec les dépressions hivernales [18] mais, en contrepartie, c'est aussi une période relativement calme comparativement à l'été. En plus, elle se caractérise par le plus faible taux de congés nous permettant ainsi d'avoir plus des participants.

Dans notre étude, nous avons utilisé l'échelle de Maslach Burnout Inventory dans sa version française. C'est l'échelle la plus utilisée actuellement pour évaluer le burnout [19]. Cependant, il n'y a pas de consensus clair en termes de nombre de dimensions à prendre en compte pour définir le burnout. En effet, certaines études emploient le terme « burnout élevé » dès qu'une seule des trois dimensions est pathologique, d'autres parlent de burnout lorsque les scores d'épuisement émotionnel ou de dépersonnalisation sont moyens ou élevés. Enfin, d'autres études entendent par « burnout modéré » des scores modérés pour chaque dimension [10, 20]. Etant donné que nous sommes dans un pays francophone, nous n'étions pas obligés de traduire l'échelle de Maslach Burnout Inventory en langue arabe.

Dans notre étude, 94,71% des participants avaient au moins une dimension pathologique. Ceux ayant au moins 2 dimensions pathologiques, soit un niveau d'épuisement professionnel modéré à élevé représentent 39,5% de la population. Ces taux sont comparables à ceux publiés dans la littérature où la prévalence varie de 25 à 50% pour les médecins [21] et de 20 à 40% pour le personnel paramédical [22]. Carla Teixeira *et al.* dans une étude portant sur 82 médecins et 218 infirmiers ont trouvé une prévalence de 31% pour les formes modérées à sévères [23]. Les mêmes constatations ont été rapportées par A. Musi *et al.* dans un travail réalisé au CHU d'Amiens en 2014 où la prévalence du burnout modéré à sévère était de 49% [24]. Une prévalence plus importante a été rapportée par une étude marocaine probablement à cause des définitions adoptées qui n'étaient pas les mêmes [17]. Ceci dit, nos résultats restent quand même inquiétants reflétant un niveau de souffrance alarmant qui peut cacher une altération profonde de la qualité de vie du personnel et de la qualité de prise en charge des malades. Ils seraient essentiellement dus à une charge de travail très importante, à des conditions d'exercice souvent insatisfaisantes et aux contraintes familiales [16]. En plus, le contexte global du pays et l'instabilité économique et administrative vécues depuis le début du printemps arabe ont altéré le bon fonctionnement des structures hospitalières rendant ainsi la tâche du personnel beaucoup plus difficile. Ils ont également contribué à la recrudescence des désordres psychiatriques dans la population générale [25]. Ces difficultés sont reflétées aussi bien par le taux alarmant du burnout que par ses répercussions graves à savoir les conduites addictives (52,65% des cas) et les idées suicidaires (4,59% des cas). De là découle un réel problème éthique puisqu'on n'a aucun accès à ces participants à haut risque suicidaire [26].

Ces répercussions ont été surtout trouvées chez les IDE de réanimation qui ont été touchés par le burnout modéré à sévère dans 72,3% des cas. Ce taux est supérieur à celui des médecins (57,15%) et des IADE (61,36%). Il pourrait s'expliquer au moins par deux raisons: c'est le sous-groupe qui connait le moins cette pathologie (12%) alors que plusieurs études confirment que plus on connait le burnout plus on est protégé [27] et en plus, cette population est plus confrontée aux situations de stress notamment celles de deuil et d'impuissance devant les impasses thérapeutiques [15, 22].

Dans la littérature, la prévalence du burnout chez les IDE varie de 15 à 40% [22]. Cette variabilité peut s'expliquer par un défaut de soutien de malades dans notre environnement, l'absence de services de développement de ressources humaines dans nos structures, le volet médicolégal qui est plus considéré dans les pays développés et l'absence de culture de divertissement pour des raisons socioéconomiques [28, 29]. Dans ce groupe qui est le plus touché, la dimension la plus altérée est l'épuisement émotionnel. Cette constatation est valable pour tous nos sous-groupes. Dans une étude faite au Portugal, la dimension la plus altérée chez le cadre paramédical est l'épuisement émotionnel [23]. Une étude brésilienne faite en 2010 portant sur 333 participants rapporte la même constatation aussi bien pour le personnel paramédical que pour le personnel médical exerçant en réanimation. Dans cette étude, les résultats sont expliqués par un argument chronologique puisque la première réaction à toute situation de stress est souvent d'ordre émotionnel [30]. Ceci souligne l'importance d'une prise en charge psychoaffective pour notre personnel. Cette prise en charge constitue à notre sens le pilier de la prévention du burnout. Elle pourrait se faire dans le cadre d'un service ou de consultation d'écoute et de suivi destiné essentiellement au personnel le plus vulnérable. Un autre rôle majeur pouvant être joué par ses structures est la formation du personnel à la gestion du stress. Ces mesures préventives nécessitent avant d'être mises en place des études sur des populations plus larges notamment dans le cadre d'une enquête nationale pouvant ainsi aboutir à des plans stratégiques [22, 31, 32].

Le syndrome d'épuisement professionnel combine une fatigue profonde, un désinvestissement de l´activité professionnelle et un sentiment d´échec et d´incompétence dans le travail. Il est considéré comme le résultat d´un stress professionnel chronique affectant tout particulièrement les professionnels de l'aide et plus spécialement le milieu d'anesthésie réanimation.

## Conclusion

Dans notre étude, uniquement 5,29% des participants ne sont pas concernés par le burnout. A la lumière de nos résultats et de ceux publiés dans la littérature, le burnout apparaît de plus en plus comme une réalité palpable chez le personnel d'anesthésie réanimation. Ces conséquences sont graves aussi bien sur le plan individuel que social. Des mesures préventives urgentes doivent être entamées idéalement dans le cadre d'un plan stratégique.

### Etat des connaissances actuelles sur le sujet

- La fréquence des problèmes de santé psychologique liés au travail augmente de façon alarmante, notamment le burnout;
- Le burnout peut altérer profondément la qualité des soins;
- Les données relatives aux pays émergents sont peu nombreuses.

### Contribution de notre étude à la connaissance

- Le burnout est particulièrement préoccupant pour le personnel d'anesthésie réanimation qui prend en charge en permanence des pathologies vitales;
- Notre étude décrit les répercussions du burnout en milieu d'anesthésie réanimation;
- Elle fournit des données spécifiques au contexte nord-africain où les contraintes socio-économiques et politiques ont connu des grandes modifications à la suite du printemps arabe.

## Conflits d’intérêts

Les auteurs ne déclarent aucun conflit d'intérêts.

## References

[1] Vézina M Preventing mental health problems linked to work: a new public health challenge. Santé Publique.

[2] Ziegel G (2015). Souffrance au travail et Burn-out. La Revue d'Homéopathie.

[3] Mealer ML, Shelton A, Berg B, Rothbaum B, Moss M (2007). Increased prevalence of post-traumatic stress disorders symptoms in critical care nurses. Am J Respir Crit Care Med.

[4] Canouï P, Canouï P (2015). Éthique et syndrome d'épuisement professionnel. Le burn-out à l'hôpital.

[5] Freudenberger HJ (1974). Staff burn-out. J Social Issues.

[6] Maslach C (1976). Burn-out. Hum Behav.

[7] Freudenberger HJ, Richelson G (1980). burnout: how to beat the high cost of success?.

[8] Larouch LM (1985). Clinical manifestations of burnout in physicians. Sante Ment Que.

[9] Maslach C, Jackson S (1981). Maslach Burnout Inventory: research edition.

[10] Maslach C, Jackson SE, Leiter MP (1996). Maslach Burnout Inventory Manual.

[11] Maslach C, Baum A, Newman S, West R, Mac Manus C (1997). Burnout in health professionals. Cambridge Handbook of psychology, Health and Medicine.

[12] Vaquin-Villeminey C (2007). Prévalence du burnout en médecine générale: Enquête nationale auprès de 221 médecins du réseau Sentinelles [Thèse].

[13] Bourée P (2015). Le burnout du professionnel de santé. Option/Bio.

[14] Massou S, Doghmi N, Belhaj A (2013). Enquête sur le syndrome d'épuisement professionnel chez les personnels d'anesthésie réanimation de quatre hôpitaux universitaires marocains. Annales Médico-psychologiques, revue psychiatrique.

[15] Chahraouia K, Bioya A, Crasa E, Gillesa F, Laurentb A, Valachec B, Quenotd JP (2011). Vécu psychologique des soignants en réanimation: une étude exploratoire et qualitative. Ann Fr Anesth Reanim.

[16] Hazif-Thomas C, Roulleaux J, Thomas P (2008). Quand la relation d'aide tombe malade, ou le travail du burnout. NPG Neurologie - Psychiatrie - Gériatrie.

[17] Doghmi N, Massou S, Balkhi H, Haimeur C, Drissi Kamili N (2013). Le burn-out, conséquences et solutions: enquête chez les personnels d'anesthésie-réanimation de quatre hôpitaux universitaires marocains. Ann Medico psychol.

[18] Chneiweiss L (2014). Le trouble affectif saisonnier. Médecine du Sommeil.

[19] Lourel M, Gueguen N (2007). Une méta-analyse de la mesure du burnout à l'aide de l'instrument MBI. L'Encéphale.

[20] Canoui P (2008). Le burnout à l'hôpital. Le syndrome d'épuisement professionnel des soignants.

[21] Embriaco N, Azoulay E, Barrau K, Kentish N, Pochard F, Loundou A, Papazian L (2007). High level of burnout in intensivists: prevalence and associated factors. Am J Respir Crit Care Med.

[22] Poncet MC, Toullic P, Papazian L (2007). burnout syndrome in critical care nursing staff. Am J Respir Crit Care Med.

[23] Teixeira C, Ribeiro O, Fonseca AM, Carvalho AS (2013). Burnout in intensive care units - a consideration of the possible prevalence and frequency of new risk factors: a descriptive correlational multicentre study. BMC Anesthesiol.

[24] Musi A, Malaquin S, Mahjoub Y, Tinturier F (2014). Prévalence du « burnout » chez les soignants de réanimation au CHU d'Amiens. Ann Fr Anesth Réanim.

[25] Ouanes S, Bouasker A, Ghachem R (2014). Psychiatric disorders following the Tunisian revolution. J Ment Health.

[26] Hoff P (2015). Suicide - ethical and juridical aspects. Ther Umsch.

[27] Mion G, Doppia MA (2014). Prise en charge des professionnels souffrant de burnout (podcast). Prat Anesth Reanim.

[28] Gillet N, Fouquereau E, Huyghebaert T, Colombat P (2016). Effets du soutien organisationnel perçu et des caractéristiques de l'emploi sur l'anxiété au travail et l'épuisement professionnel: le rôle médiateur de la satisfaction des besoins psychologiques. Psychologie Française.

[29] Ott JC (2011). Government and happiness in 130 nations: Good governance fosters higher level and more equality of happiness. Soc Indic Res.

[30] Tironi MO, Nascimento Sobrinho CL, Barros Dde S (2009). Professional Burnout Syndrome among Intensive Care Physicians in Salvador, Bahia, Brazil. Rev Assoc Med Bras (1992).

[31] Bonnet F, Dureuil B (2011). Prévenir le syndrome d'épuisement professionnel en anesthésie-réanimation. Ann Fr Anesth Reanim.

[32] Lederer W, Kinzl JF, Trefalt E, Traweger C, Benzer A (2006). Significance of working conditions on burnout in anesthetists. Acta Anaesthesiol Scand.

